# Supplementary material for: Distinct contributions of tensile and shear stress on E-cadherin levels during morphogenesis
Source: Nat Commun. 2018 Nov 27;9:5021. doi: 10.1038/s41467-018-07448-8 (PMC6258672; doi:10.1038/s41467-018-07448-8)
Supplement: Supplementary file 1 — Supplementary Information [file 41467_2018_7448_MOESM1_ESM.pdf]

Supplementary information for

Distinct contributions of tensile and shear stress on E-cadherin levels during morphogenesis

Kale et al.

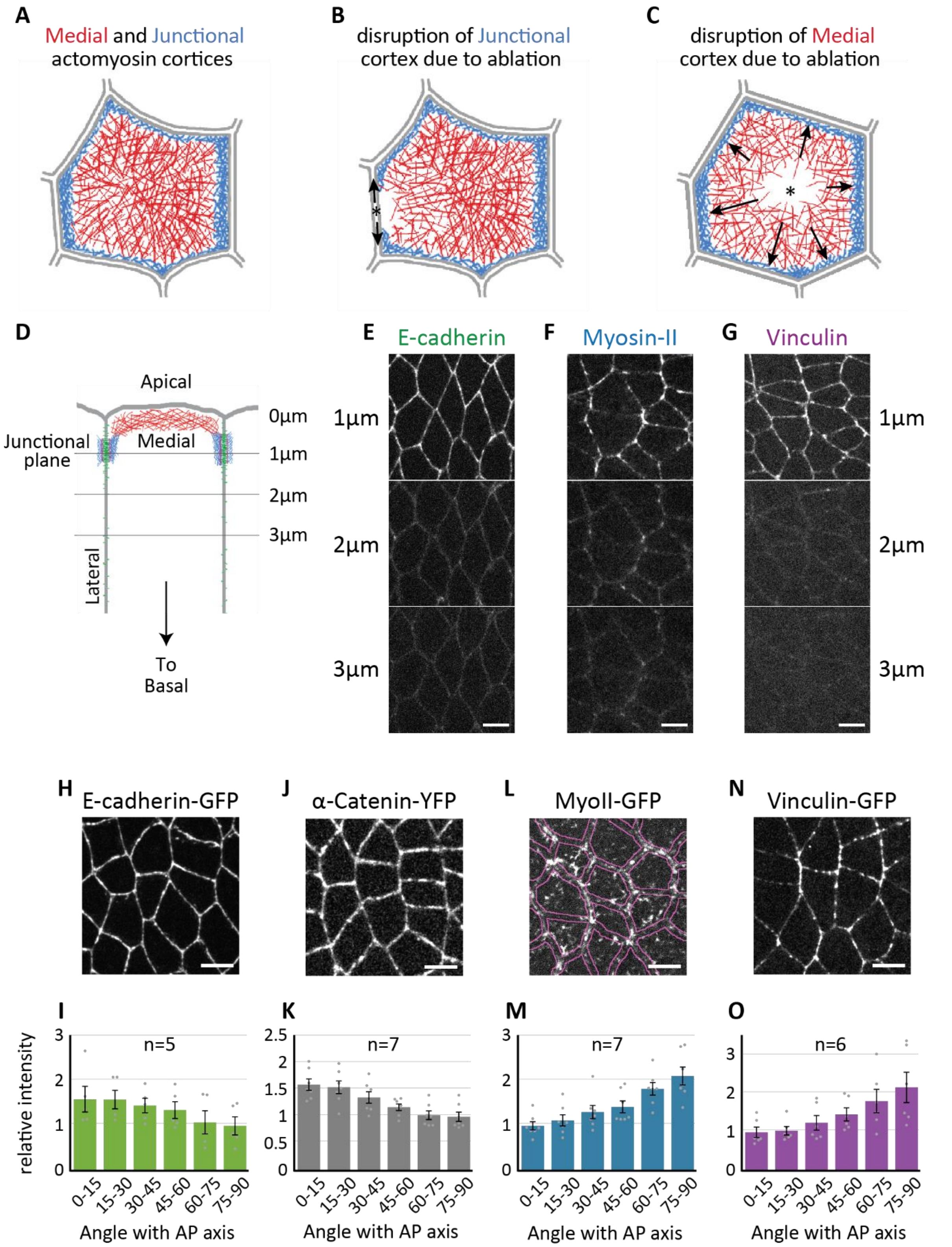

Supplementary Figure 1: Cellular sources of tension and the distribution of Vinculin in the embryonic ectoderm

Schematic in A shows the medial and junctional actomyosin cortices. Contractions in the junctional Myosin-II tries to maintain junctions under parallel tension, which can be released using junctional ablations (B) to see a tension relaxation and a consequent recoil of the vertices. Contractions in the medial cortex pull on the junctions in a perpendicular direction

and can produce inward curvature, which can be released using medial ablation (C) to see a loss of said curvature.
Apico-basal polarity: (D) Schematic representation of apical region of the ectodermal cells. Various horizontal lines correspond to cross-sections shown in E, F, and G. (E-G) Planar view of E-cadherin, Myosin-II and Vinculin at different z-steps of 1µm from apical surface. E-cadherin, Myosin-II and Vinculin enrichment in junctional plane (1µm depth) is evident.
Planar polarity: (H, J, L, and N) Images showing z-projection over 3µm apical region to represent junctional localization of E-cadherin (H), α-Catenin (J), Myosin-II (L) and Vinculin (N). Boundary between cytoplasm and cell contacts is emphasized in L, as Myosin-II distribution doesn't mark all junctions. (I, K, M, and O) Planar polarity quantifications for E-cadherin (I), α-Catenin (K), Myosin-II (M) and Vinculin (O). Number of embryos in insets. Error bars represent SEM.

All scale bars are 5µm. Images/quantifications in E, H, and I come from embryos expressing E-cadherin-GFP. Images/quantifications in F, L, and M come from embryos expressing MyoII-GFP. Images/quantifications in G, N, and O come from embryos expressing Vinculin-GFP. Images/quantifications in J and K come from embryos expressing α-Catenin-YFP.

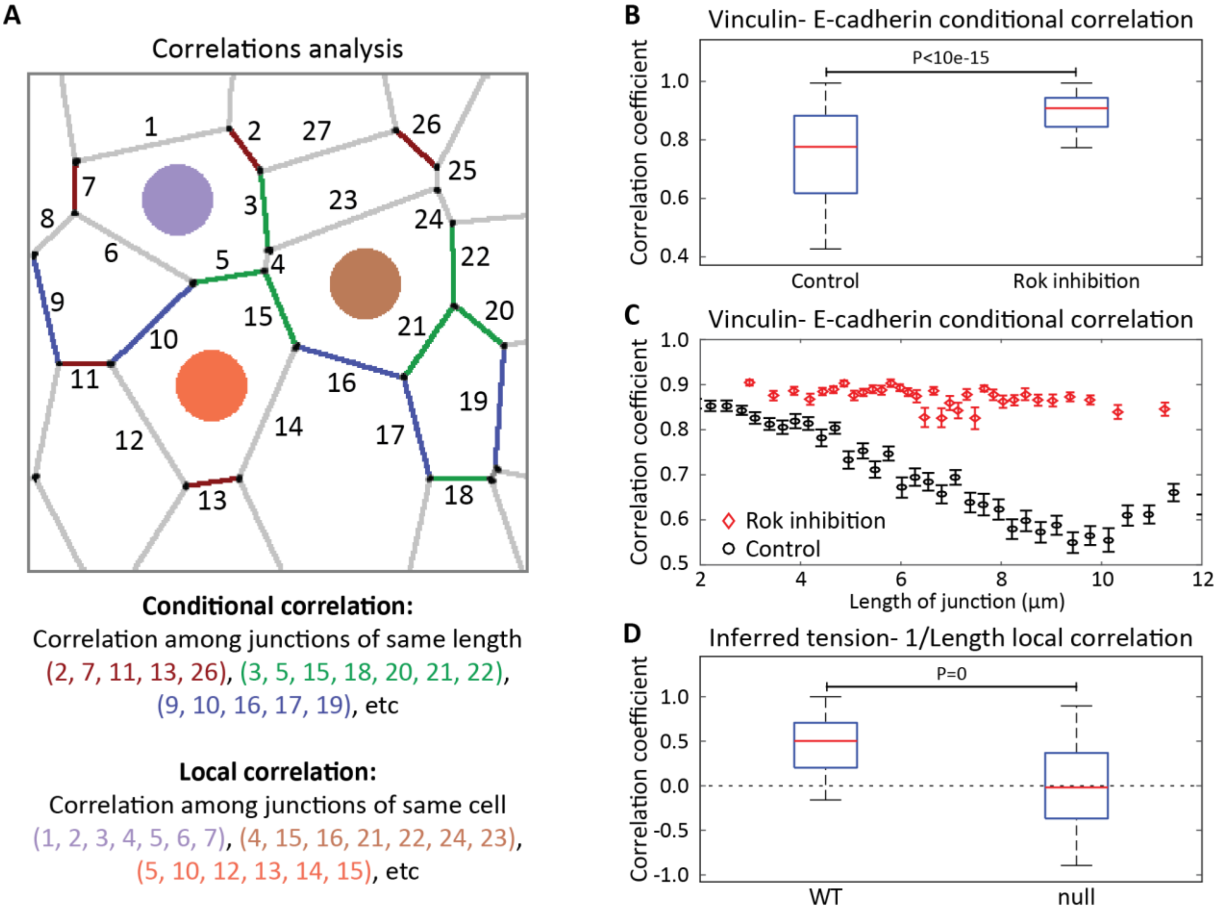

Supplementary Figure 2: Additional correlation analyses

(A) Schematic representation to describe ‘conditional correlation’ and ‘local correlation’. Various junctions are numbered as shown. Similarly colored junctions belong to the same length category and will be binned together for ‘conditional correlation’ analysis. Junctions of the same cell will be binned together for ‘local correlation’ analysis. Also, see Methods.

(B, C) Conditional correlation of Vinculin intensity with E-cadherin intensity conditioned on the length of the junction for wild-type and Rok inhibited embryos. The statistics are based on 35180 junctions across 6 embryos with 10 junctions in one conditional bin for both wild-type and Rok inhibited embryos.

(D) Box plots showing the distribution of local correlation coefficient between inferred tension and the inverse of junction length. The statistics are based on 9000 cells across 6 embryos.

The boxes in B and D represent 25th percentiles to 75th percentiles; the whiskers represent 5th percentiles to 95th percentiles; and the red lines represent the medians. The error bars in C represent the standard error across 100 different bins with the same length of junction. Vinculin- E-cadherin correlations in B and C were estimated for appropriately injected embryos co-expressing Vinculin-mCherry and E-cadherin-GFP.

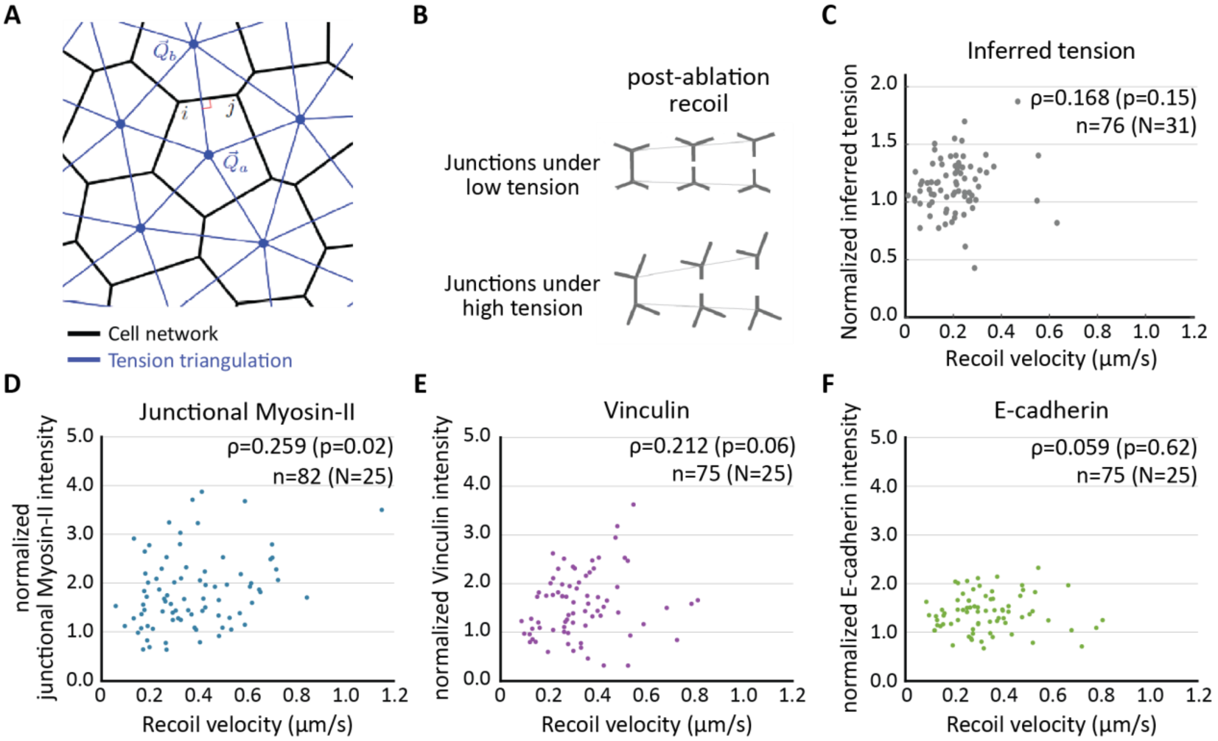

Supplementary Figure 3: Additional ablation and mechanical inference analyses
(A) Schematic representation for use of tension triangulation to estimate inferred tensions from cellular networks. See 'Methods: mechanical inference' for more details.
(B) Schematic representation of junctional recoil after an event of ablation. Junctions under low tension (top panels) show slower initial recoil, whereas junctions under high tension (bottom panels) show faster initial recoil. See 'Methods: laser ablations' for more details
(C-F) Scatter plots showing the distribution of the pre-ablation inferred tension (C), junctional Myosin-II intensities (D), Vinculin intensities (E), or E-cadherin intensities (F) plotted against the post-ablation recoil velocities for multiple events of junctional ablations. In insets, Spearman correlation coefficient 'ρ', corresponding 'p-value', and number of ablations events 'n', pooled from 'N' embryos.

Quantification in D come from embryos expressing MyoII-GFP. Quantifications in E and F come from embryos co-expressing Vinculin-mCherry and E-cadherin-GFP, which is the same set of ablation events as those in Figure 2E. Quantifications in C are pooled subsets of two ablation data sets on which mechanical inference could be performed; first, same as E and F above (n=54, N=18), and second the 'controls' used in Supplementary Figure 4A (n=22, N=13).

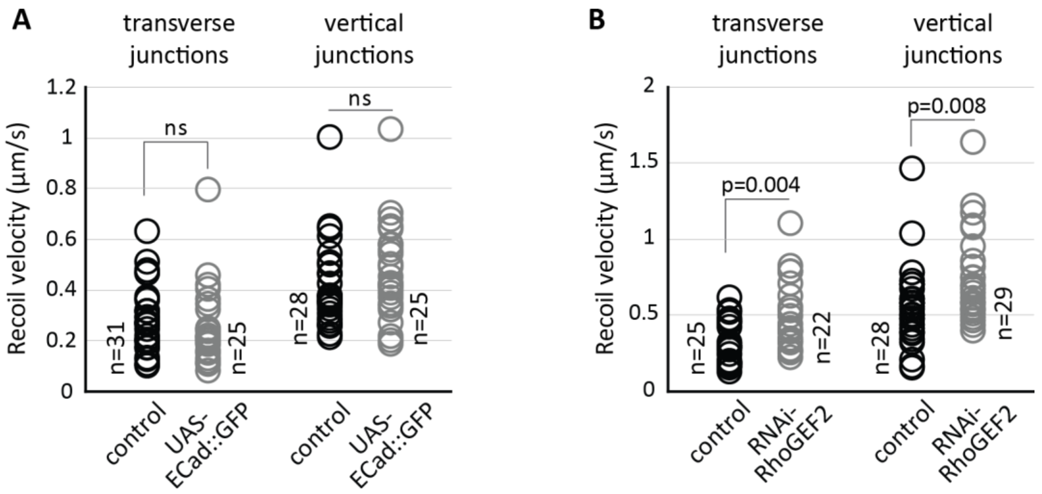

Supplementary Figure 4: Ablation analyses to estimate the distribution of junctional tension

(A) The distribution of recoil velocities didn't change in either the transverse or vertical junctions upon E-cadherin over-expression, indicating that the distribution of junctional tension hasn't changed either. Number of ablated junctions in each category is indicated next to its distribution. Transverse and vertical junctions pooled from various ablation events in 17 embryos for control and 9 embryos for UAS-E-cad::GFP

(B) The recoils are faster upon RhoGEF2-RNAi, in both the transverse and vertical junctions, indicating that the junctional tension hasn't decreased. Number of ablated junctions in each category is indicated next to its distribution. Transverse and vertical junctions pooled from various ablation events in 14 embryos for control and 9 embryos for RhoGEF2-RNAi.

Statistical significance estimated using 'Mann-Whitney U-test'. Quantifications in 'A' come from embryos co-expressing MyoII-mCherry and E-cadherin-GFP, with or without accompanying E-cadherin over-expression. Quantifications in 'B' come from embryos expressing MyoII-GFP, with or without accompanying RhoGEF2-RNAi.

ns, $p>0.05$

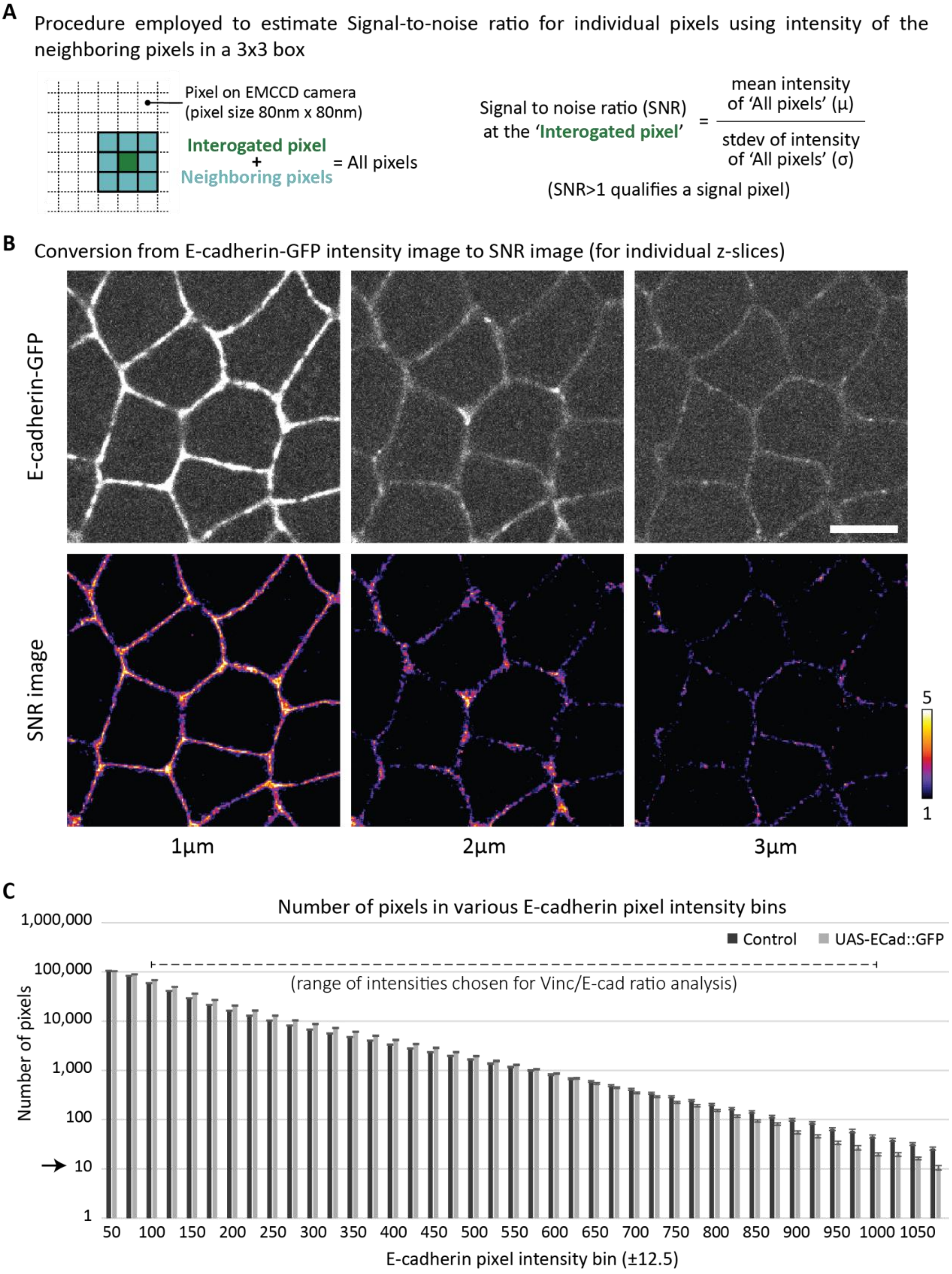

Supplementary Figure 5: Supporting information for 'pixel analysis' of Vinc/E-cad ratio
(A) Schematic representation of the concept employed to identify pixels positive for E-cadherin. Signal-to-Noise Ratio (SNR) provides a completely objective way to determine a signal pixel. SNR is estimated using a simple local estimation of the ratio between the mean

(μ) of pixel intensities and the standard deviation (σ) of pixel intensities. At the signal pixel, the ratio of pixel intensities from Vinculin and E-cadherin image channels gives the Vinc/E-cad ratio.
(B) Representative images showing the conversion from E-cadherin channel image to SNR image. The E-cadherin-GFP images are at 1, 2 or 3μm distance from the surface of the embryo (similar to Supplementary Fig. 1D). The corresponding SNR images show all pixels with intensity>1. In such a representation, all cytoplasmic pixels are blank. Scale bar 5μm.
(C) Quantifications showing the number of pixels in various E-cadherin pixel intensity bins. Each bin is 25 intensity units wide. The number of pixels is comparable between control and E-cadherin over-expressing embryos, across an order of a magnitude of pixel intensities. Number of pixels in a bin are separately estimated for individual embryos. Mean and SEM are calculated across embryos. All error bars represent SEM. Arrow indicates the minimum average number of pixels required to have meaningful statistics for individual embryos in both control and E-cadherin over-expressing embryos.

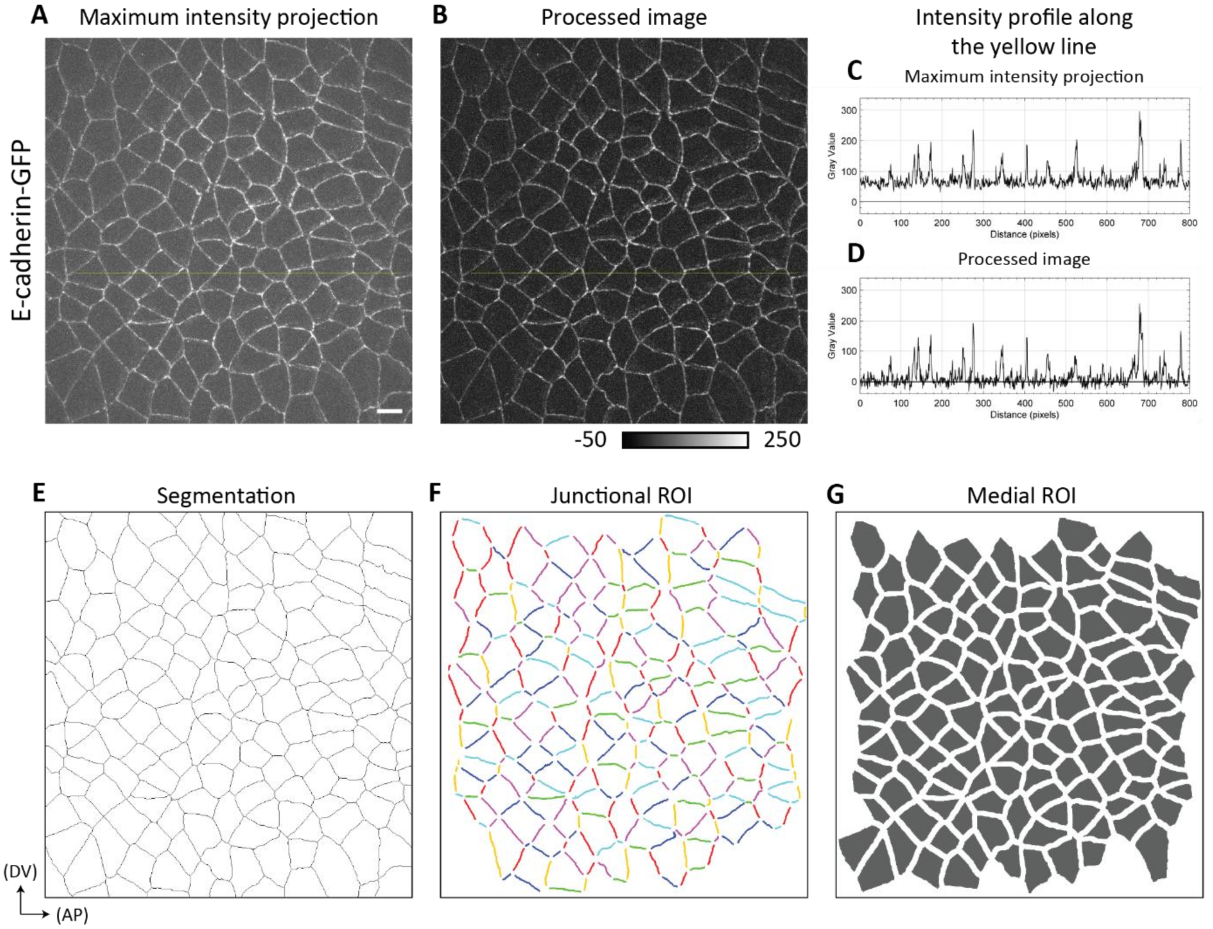

H Follow-up from C′: Segregate 'line density' values (ρ) for junctions (j) based on their angle relative to the AP axis for each embryo (E). Using that, calculate 'averaged line density' (ALD) and 'planar cell polarity' (PCP) for each of 'N' embryos. These values are used to calculate 'mean junctional intensity' and 'amplitude of polarity', as shown. The same values are used to estimate the related SEM values.

Angle bins →	(AP) 00-15	15-30	30-45	45-60	60-75	(DV) 75-90
	$\rho(j_a)$	$\rho(j_c)$	$\rho(j_d)$	$\rho(j_f)$	$\rho(j_g)$	$\rho(j_h)$
	$\rho(j_b)$	$\rho(j_i)$	$\rho(j_e)$	$\rho(j_j)$	$\rho(j_l)$	$\rho(j_m)$
	$\rho(j_p)$	$\rho(j_o)$	$\rho(j_t)$	$\rho(j_k)$	$\rho(j_w)$	$\rho(j_n)$
	$\rho(j_q)$	$\rho(j_r)$	.	$\rho(j_u)$	$\rho(j_y)$	$\rho(j_x)$
	$\rho(j_s)$	.	.	$\rho(j_v)$	.	$\rho(j_z)$
	.	.	.	.	.	.
Averages for each categories →	$Avg_{(00\text{-}15)}$	$Avg_{(15\text{-}30)}$	$Avg_{(30\text{-}45)}$	$Avg_{(45\text{-}60)}$	$Avg_{(60\text{-}75)}$	$Avg_{(75\text{-}90)}$

$ALD(E_1) = (Avg_{(00\text{-}15)} + Avg_{(15\text{-}30)} + ... + Avg_{(75\text{-}90)})/6$ ⟶ mean junctional intensity $= (ALD(E_1) + ALD(E_2) + ... + ALD(E_N)) / N$

$PCP_{DV/AP}(E_1) = Avg_{(75\text{-}90)}/Avg_{(00\text{-}15)}$ ⟶ amplitude of polarity$_{DV/AP} = (PCP_{DV/AP}(E_1) + PCP_{DV/AP}(E_2) + ... + PCP_{DV/AP}(E_N)) / N$

I Follow-up from C′′: Pool 'area density' values (R) for 'n' number of cells per image. Using that, calculate 'averaged area density' (AAD) for each of 'N' embryos (E). These values are used to calculate 'mean medial intensity', as shown. The same values are used to estimate the related SEM values.

$AAD(E_1) = (R_1 + R_2 + ... + R_n) / n$ ⟶ mean medial intensity $= (AAD(E_1) + AAD(E_2) + ... + AAD(E_N)) / N$

Supplementary Figure 6: Supporting information for image analysis workflow

Here we are giving an example to show various steps for the image analysis to quantify junctional and medial intensities.

<u>'Image processing workflow' sets the background intensities to zero:</u> (A, B) Example images showing E-cadherin intensity distribution in a 'Maximum intensity projection' (A) of the raw data and a 'processed image' (B) that is obtained at the end of image processing part of the workflow. Note that the contrast between A and B is the same and is shown in LUT bar below

B. The intensity profiles along the yellow lines are presented in C and D. As it can be clearly seen, image processing shifts the baseline intensity values close to zero, without affecting the heights of individual peaks. Thus, the contribution from the background to junctional/ medial intensity measurements is only minor, if any.
‘Segmentation workflow produces ROIs for cell contacts (junctional pool) and cells (medial pool): (E) Example image showing the segmentation output for example image presented in A. As described in Methods, cell contacts are first segmented using ‘Packing Analyzer v2.0’ and then manually corrected to ensure accuracy. Using this segmentation, junctional ROIs (F) and medial ROIs (G) are automatically generated using a custom written macros in FIJI, which are further corrected manually to ensure accuracy.
(H) Here, we are giving a visual representation of how the junctional intensities are processed to get ‘mean junctional intensity’. This is a follow-up of ROIs presented in F and uses another custom written FIJI macro.
(I) Here, we are giving a visual representation of how the medial intensities are processed to get ‘mean medial intensity’. This is a follow-up of ROIs presented in G and uses a separate custom written FIJI macro.
